# Evaluation of Impacted Mandibular Third Molars by Panoramic Radiography

**DOI:** 10.5402/2011/406714

**Published:** 2010-09-26

**Authors:** Siddharth Gupta, Rahul R. Bhowate, Nitin Nigam, Sonal Saxena

**Affiliations:** ^1^Department of Oral Medicine and Radiology, Career Institute of Dental Science and Hospital, Sitapur-Hardoi Bypass, Dubagga, Lucknow, India; ^2^Department of Oral Medicine and Radiology, Sharad Pawar Dental College, Sawangi (Meghe), Maharashtra, Wardha, India; ^3^Smile Care Dental Clinic, Purani Bazar, Karwi, Chitrakoot, UP, India

## Abstract

*Objective*. The study was to evaluate impacted mandibular third molars (IM3M) for their angulation, level of eruption, third molar space and relation of inferior alveolar canal with their roots. *Methods*. Total 988 IM3M were studied in 578 individuals of age 18 years and above, dividing them into three groups i.e. symptomatic, asymptomatic and radiographic only. Individuals were also divided according to age, sex and side of IM3M (right or left). Panoramic radiographs were obtained after written consent and traced. *ℵ* ^2^-test was applied to check inter-group and intra-group significance. *Result*. Out of 578 individuals 307 (53.11%) were males and 271 (46.89%) females. Maximum number of IM3M were in 18-27 years age group (398 i.e. 68.89%). Out of 988 IM3M, 39.93% were vertically placed. 61.84% IM3M were found at level A. Class II (79.65%) was the most common relation for third molar space. Notching (12.55%) was most common true inferior alveolar canal and IM3M root relation whereas superimposed (41.80%) was most common false inferior alveolar canal and IM3M root relation. For all the criteria significant inter-group difference was found (considering *P* < .05) and intra-group difference was non significant. *Conclusion and significance*. Panoramic radiographs can be used as reliable investigation for evaluation of IM3M.

## 1. Introduction

The removal of impacted third molars is the most common procedure in the specialty of Oral and Maxillofacial surgery. The procedure can be simply performed using elevators and/or forceps, but may require surgical intervention. This increases the risk of complications, such as nerve paresthesia, alveolar osteitis, hemorrhage, or even fracture of the jaw [1]. The reported frequency of inferior alveolar canal injury associated with mandibular third molars removal ranges from 0.6% and 5.3%. The risk of permanent inferior alveolar canal injury is less than 1% [2]. To some extent these complications can be anticipated prior to surgery by using radiographs, which can help surgeon to take steps to avoid or inform the patient of the likelihood of their occurrence. 

Currently, the panoramic radiograph is the technique of choice to evaluate impacted mandibular third molars. The estimated sensitivity for radiographic signs, as predictor of nerve injury ranges from 24% to 38%, and the specificity ranges from 96% to 98% [3]. In this way, panoramic radiography permits an initial evaluation of any problems related to impacted mandibular third molar [4]. 

After the approval from Institutional Ethical Committee of Datta Meghe Institute of Medical Sciences Deemed University, the present study was undertaken to evaluate the status of impacted mandibular third molars in the rural population of Wardha district, attending Outpatient Department of Oral Medicine and Radiology, Sharad Pawar Dental College & Hospital, Sawangi (M), Wardha, India. All the impacted mandibular third molar included in the study were evaluated for type of angulation, level of eruption (depth of impaction), available space and relation of their root with inferior alveolar canal on the basis of panoramic radiographic presentation, also the intergroup and intragroup relation was evaluated for any significant difference.

## 2. Subjects and Method

The present study consisted of 578 subjects; they were divided into three main categories: Asymptomatic group (289), Symptomatic group (241), and Radiographic group (48). These groups were further divided according to findings of right and left sides of mandibular third molars, age, and sex. Patients over 18 year of age visiting the Department of Oral Medicine and Radiology were evaluated. Patients were divided into three age groups. Age range of first group was 18 years to 27 years, age range of second group was 28 years to 37 years, and age range of third group was 38 years and above. In asymptomatic Group or Group I (Third molar/molars without clinical Symptoms), patients without any complaint (previous or present) associated with clinically visible mandibular third molar (unilaterally or bilaterally) were included. In symptomatic Group or Group II (Clinically symptomatic Third molar/molars), patients having complaint or gave history of complaint associated with clinically visible mandibular third molars (unilaterally or bilaterally) were included and in radiographic Group or Group III (Clinically no evidence of Third molar/molars), patients who had been radiographed for other condition/complaint, but showing impacted mandibular third molar/molars were included in this group, patients other than group I and II. 

Exclusion criteria: any patient with history of extraction of permanent tooth, mandibular fracture, or orthodontic treatment was excluded from the study, also patients with developmental anomaly, congenital or systemic disease and/or major pathology in the mandible that has/had caused severe bone resorption/destruction, bone expansion, root resorption, and tooth migration were excluded from the study. Also third molars having underdeveloped roots (radiographically third molars having less than two-third root formation) were excluded [2], and considered as underdeveloped.

**Figure 1:**
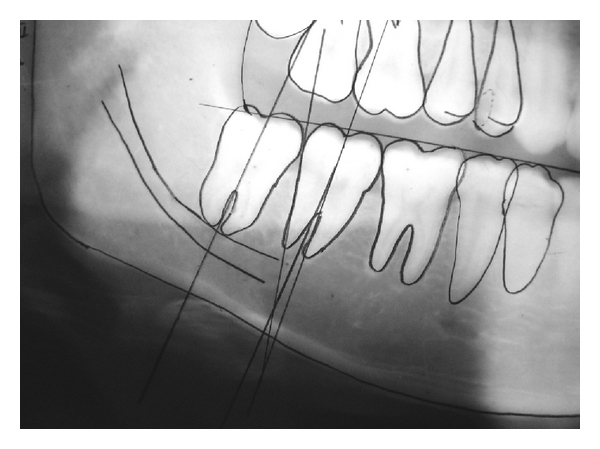


Patients were examined clinically under aseptic condition and informed consent was obtained. Radiographs were taken according to Panoramic Machine (Planmeca Proline CC Panoramic X-ray, Planmeca OY Helsinki, Finland), specification, which has a constant magnification of 1.2. Exposed panoramic films were processed manually by visual inspection method. Outline of the lower border of mandible, mandibular condyle and coronoid, anterior and posterior border of ramus of the mandible along-with all the first molars were traced as reference point. Outline of mandibular first premolar, second premolar, first molar, second molar and third molar of right and left sides were traced. Following Ganss Method [5], occlusal plane was drawn through the tip of the most superior cusps of the first premolar and the tip of the most superior mesial cusps of second molar, extending up to anterior border of the ramus of the mandible. A perpendicular line was drawn from the occlusal plane touching the most distal point of the second molar (Figure 1). 

The available third molar space was determined as the distance between the intersection of the occlusal plane with the anterior border of the ramus and the intersection of the vertical line with the occlusal plane. Also the mesiodistal width of the third molar crown was recorded. If the available space is more or equal to mesiodistal diameter of third molar, it was considered as Class I (adequate room for eruption of a third molar if eruption could occur), if the available space was less than mesiodistal diameter of third molar it was considered as Class II (partial space between posterior of the second molar and the ascending ramus of the mandible), and if the tooth was located completely within the mandibular ramus it was considered as Class III) the retromolar space is obliterated because the ascending ramus of the mandible was located immediately posterior to the second molar) [6, 7]. 

Level of eruption was recorded level A when there was crown to crown position between impacted third molar and second molar, level B when there was crown to cervical position between impacted third molar and second molar, and level C when there was crown to root position between third molar and second molar [2, 6–9]. 

The inclination of third molars was determined by measuring the angle formed between the line intersecting the long axis of the second and third molars, drawn through the midpoint of the occlusal surface and midpoint of the bifurcation [2, 6]. Inclination of third molars was considered vertical if angle was ±10°, mesioangular if angle was +11° to 70°, distoangular if angle was –11° to –70°, and horizontal if more than 70° [2]. (“+ sign” denotes that intersection of line is above the molars and “– sign” denotes that intersection of line is below the molars.). 

Outline of inferior alveolar canal was traced to record its relation with third molar roots as in [10].

1. When superior border of canal was touching the roots apices or within 2 mm below them, relation considered was Adjacent.
2. When the canal was superimposed over part of the roots which appeared less radiopaque than the remaining radiological image of the roots, relation considered was Superimposed.
3. When there was a radiolucent band at the apex of the roots, a break in the continuity of the upper radio dense border, and narrowing at the expense of the top of the canal was present, relation considered was Notching.
4. The relation was considered as Grooving, when radiolucent band across the root above apex was present with interruption of both superior and inferior borders of the canal, and narrowing of the canal space.
5. With radiolucent band crossing the root above the apex and loss of both superior and inferior borders of the canal at the area where they cross the root, with constriction of the canal maximal in the middle of the root was present, relation considered was Perforation.
6. When there was no relation between the canal and the root apices, condition was recorded as None.

Notching, grooving and perforation were regrouped as true relation. Superimposed, adjacent, none were regrouped as false relation [10]. *ℵ* ^2^-test was used to check intragroup and intergroup significance. Kappa test was applied to check intraobserver reliability after retracing 10 panoramic radiographs from each group.

## 3. Result

Out of 1156 mandibular third molar sites evaluated, 988 (85.47%) mandibular third molars were evaluated in the study. Remaining mandibular third molars were missing, underdeveloped, or erupted. All the mandibular third molars included in the study, irrespective of their group were evaluated and classified as age-wise and sex wise distribution of patients, clinical presentation, and sidewise distribution of patients, angulation-wise distribution of mandibular third molars, depth-wise distribution of mandibular third molars, third molar space-wise distribution of mandibular third molars, and distribution of mandibular third molar according to its relation with inferior alveolar canal.

Out of total 578 subjects, 307 (53.11%) were males and 271 (46.89%) were females. Mean age of the total male patients was 26.49 (±7.48) years. Mean age of the total female patients was 25.02 (±6.55) years (Table 1).

Out of 1156 sites 1139 mandibular third molars were evaluated as 117 mandibular third molars were erupted (right side 72 and left side 45). Out of 1139 mandibular third molars, 780 (75.07%) mandibular third molars were partially erupted and 259 (24.92%) mandibular third molars were not erupted. There was highly significant difference between the eruption status of different groups (considering *P* < .0001). No significant difference between the eruption statuses was found when right side and left side was compared (Table 2).

After combining all the groups, a total of 1039 sites were evaluated. Out of that, 988 impacted mandibular third molars were included in the study as 39 mandibular third molars were missing and 12 were underdeveloped. A total of 355 (35.93%) impacted mandibular third molars were mesioangular, 151 (15.28%) impacted mandibular third molars were distoangular, 93 (9.41%) impacted mandibular third molars were horizontal, and 389 (39.37%) impacted mandibular third molars were vertical. There was significant difference between the angulations of different groups (considering *P* < .05). No significant difference between the angulation was found when right and left sides were compared (Table 3).

Out of 988 impacted mandibular third molars included in the study 611 (61.84%) impacted mandibular third molars were at level A, 321 (32.48%) impacted mandibular third molars were at level B, and 56 (5.66%) impacted mandibular third molars were at level C. There was highly significant difference between the level of eruption of third molar in different groups (considering *P* < .0001). No significant difference between the level of eruption was found when right and left sides were compared (Table 4).

Out of 988 impacted mandibular third molars, 180 (18.21%) impacted mandibular third molars were in class I relation, 787 (79.65%) impacted mandibular third molars were in class II relation, and 21 (2.12%) impacted mandibular third molars were in class III relation. There was significant difference between the third molar spaces in different groups (considering *P* < .05). No significant difference between the third molar spaces was found when right and left sides were compared (Table 5).

In 211 (21.35%) true relations a total of 124 (12.55%) relations were notching, 67 (6.78%) relations were grooving, and 20 (2.02%) relations were perforation. In 777 (78.64%) false relations a total of 413 (41.80%) relations were superimposed, 275 (27.83%) relations were adjacent and in 89 (7.69%) cases there were no relations. There was significant difference between the third molar roots and inferior alveolar canal relation in different groups (considering *P* < .05). No significant difference between inferior alveolar canal relations was found when right and left sides were compared (Table 6).

Kappa agreement for different variables ranged from moderate to perfect.

## 4. Discussion

A total of 578 samples were included in the present study, and 1156 mandibular third molar sites were evaluated clinically. The maximum number of samples (398, i.e., 68.86%) were in age group of 18–27 years, studies by Sandhu and Kapila, Chiapasco et al., Hazza'a et al. also showed maximum no of subjects in similar age group [7, 10, 11]. Many impacted third molars can change their position and erupt by the middle of the third decade. This indicates that the eruption period for third molars is longer than supposed previously. Unerupted teeth can continue to change position after skeletal growth is complete and the tooth is fully formed. Insufficient information exist to clearly define when in an individual, permanent tooth will remain unerupted. Virtually all horizontally impacted teeth, teeth in vertical ramus and those unerupted by middle of third decade are considered to remain impacted [12].

Out of 578 samples of present study, 307 (53.11%) were males and 271 (46.89%) were females. For gender distribution this study is in accordance with study of Hazza'a et al. [10] However, studies of Linden et al., Hattab et al., Yamaoka et al., Sandhu and Kapila, and Odusanya and Abayomi showed female predominance [11, 13–16]. This lack of definitive sex predominance in the third molar impaction raised the question against Hellmen's statement that the jaws of the females stop growing when third molar just begin to erupt, whereas in males the growth of the jaws continues beyond the time of third molar [14].

Eruption status wise distribution of mandibular third molars of present study showed maximum number of partially erupted third molars (75.07%) followed by unerupted (including missing and underdeveloped) mandibular third molars (24.92%). Results of the present study are in accordance with that of an Indian study of Sandhu and Kaur [2]. Present study is also in agreement with study of Knutsson et al. [17]. Results of Ventä et al. [18] were not in agreement with present study as they found maximum number of unerupted mandibular third molars followed by partially erupted mandibular third molars at the age of 20 years. This may be because of their restricted age sample or due to population difference. 

Highest number of mandibular third molars were in vertically position (389, i.e., 39.37%), followed by mesioangular, distoangular, and horizontal position. Results of present study is in accordance with the study of Hazza'a et al. [10] as they also found highest number of vertically placed third molars followed by mesioangular, distoangular, and horizontal third molars. Rajasuo et al. [19] also found highest number of vertically placed third molars in their study. Number of mesioangular third molars in present study are in accordance with the study carried by Valmaseda-Castellon et al. [20], as they found 358 mesioangular mandibular third molars in a total of 1000 teeth they evaluated, but result was not in agreement for vertically placed, distoangular, and horizontally placed third molars. Linden et al., Hattab et al., Knutsson et al. and Sedaghatfar et al. in their study found maximum number of third molars to be mesioangular [3, 13, 14, 17]. In study of Richardson [21] he found maximum number of third molars in horizontal position. In another study by Chu et al. [22], they found that maximum number of third molars (80% of 3178 mandibular third molars) were horizontal or mesioangular. These variations in angular position of mandibular third molars may be because of the fact that the studied population in each study was quite different from each other. 

Present study shows maximum number of third molars at level A (611, i.e., 61.84%), followed by level B (321, i.e., 32.48%) and level C (56, i.e., 5.66%). Level of eruption in the present study is in agreement with that of Jerjes et al. [1] and also with study of Hattab et al. [14]. Study of Sandhu and Kaur, Susarla and Dodson found maximum third molars at level B followed by level A and level C [2, 23].

As maximum number of mandibular third molars in the present study are partially erupted (67.48%), it was found that 787 (79.65%) mandibular third molars are in class II relation, followed by 180 (18.21%) in class I and 21 (2.12%) in class III. Results of present study are in accordance with that of Susarla and Dodson [23] as they also found maximum third molars in class II relations followed by class I and class III relations. Results were not in agreement with that of Jerjes et al. [1] as they found maximum number of mandibular third molars in class I relation followed by class II and class III. An important variable to predict the eruption of third molar is mesiodistal space, measured from a panoramic radiograph. Lack of space seems to be major cause of abortive eruption. However eruption cannot be guaranteed, despite adequate space available in the jaw [2]. Hattab and Abu Alhaija [6] reported that the space behind the second molar was reduced in 90% of cases with mandibular third molar impaction. Radiographic techniques used to assess lower third molar space and mandibular linear dimensions and angles's panoramic radiography yielded one of the most accurate estimations [6, 24]. Lack of space is single most important cause of impaction of third molars. The average space/crown width ratio was 1:1 for erupted group and 0.8 for the impacted group [6]. But according to Ventä et al. [2, 25]. It may be inaccurate to predict the eruption of third molars before the age of 20 years because of continuously positional changes of the third molars during further development. 

The present study showed that the inferior alveolar canal relation in maximum number of third molars was superimposed (413, i.e., 41.80%), followed by adjacent (275, i.e., 27.83%), notching (124, i.e., 12.55%), no relation/none (89, i.e., 9.00%), grooving (67, i.e., 6.78%), and perforation (20, i.e., 2.02%). The categories notching, grooving, and perforation were regrouped together and called true relationship (211, i.e., 21.35%), and categories superimposed, adjacent, and none were regrouped together and called false relationship (777, i.e., 78.64). Hazza'a et al. [10] also found maximum number of relations to be superimposed (45.5%), followed by adjacent (26.2%), but results of present study did not match for other relations as they found no relation (12.6%) to be the third highest, followed by grooving (12.35%), notching (3.0%), and perforation (0.35%). Results of present study for true relation and false relation are in accordance with that of Hazza'a et al. [10] as they also found 15.5% true relations and 84.5% false relations. 

In the present study angulation-wise distribution of inferior alveolar canal relation showed maximum mesioangularly placed third molars with superimposed relations (182, i.e., 18.42%), followed by vertically placed third molar with superimposed relations (146, i.e., 14.77%), then vertically placed third molar with adjacent inferior alveolar canal relations (112, i.e., 11.33%) and mesioangularly placed third molars with adjacent relations (82, i.e., 8.30%). Present study is not in agreement with study of Hazza'a et al. [10] as they found maximum number of vertically placed third molars with superimposed relations, followed by vertically placed third molars with adjacent relations, then mesioangularly placed third molars with superimposed relations and mesioangularly placed third molars with grooving relations.

In the present study, 381 (32.48%) level B and 56 (5.66%) level C impacted mandibular third molars have less chance to erupt into the oral cavity, as they are in the mean age group of 24.41 to 26.79 (±6.27) years. These level B and C teeth are required to be observed in their eruption process, and those of symptomatic will require surgical removal. 611 (61.84%) of third molars may erupt in the oral cavity as they were at the occlusal level of second molar, if there is no any other obstruction like dense soft tissue/bony covering, reduced third molar space or mesioangular, distoangular and horizontal position of the third molar.

From the present study it is concluded that only 180 (18.21%) of third molars had sufficient space for eruption and may erupt. 787 (79.65%) showed increased mesiodistal width of crown when compared to the space available between distal to second molar and anterior border of ramus of the mandible, 21 (2.12%) impacted mandibular third molars had no chance of eruption from the radiographic analysis as there was no space available, these teeth will not erupt in the oral cavity and should be followed. In the present study 389 (39.37%) mandibular third molars had vertical position which had 9.1% true relation and 30.14% false relations, compared to mesioangular position which showed 7.11% true and 28.74% false relation. True relations, that is, notching, grooving, and perforation were highest in vertically placed third molars followed by mesioangular impaction. These findings suggest that a surgeon should be careful even while performing disimpaction of vertically or mesioangularly placed third molars to avoid any injury to inferior alveolar canal. 

In conclusion panoramic radiograph can be used as a valuable predictor of outcome of the impacted mandibular third molars position, as they appear to have quite good cost-information ratio.

## Figures and Tables

**Table 1:** Agewise and sexwise distribution of total patients.

Age Group (yrs)	Group I		Group II		Group III		Total		Grand Total
	Male	Female	Male	Female	Male	Female	Male	Female	
18–27	100 (17.30%)	107 (18.51%)	82 (14.19%)	73 (12.63%)	17 (2.94%)	19 (3.29%)	199 (34.43%)	199 (34.43%)	398 (68.86%)
28–37	33 (5.71%)	26 (4.50%)	38 (6.57%)	28 (4.84%)	4 (0.69%)	3 (0.52%)	75 (12.98%)	57 (9.86%)	132 (22.84%)
38 and above	17 (2.94%)	6 (1.04%)	14 (2.42%)	6 (1.04%)	2 (0.35%)	3 (0.52%)	33 (5.71%)	15 (2.60%)	48 (8.30%)
Total	150 (25.95%)	139 (24.05%)	134 (23.18%)	107 (18.51%)	23 (3.98%)	25 (4.33%)	307 (53.11%)	271 (46.89%)	578 (100.0%)
Mean Age	26.49	24.41	26.79	25.71	24.91	26.0	26.49	25.02	25.82
SD	7.54	6.27	7.38	6.18	7.63	9.28	7.48	6.55	7.10
Min	18	18	18	18	18	18	18	18	18
Max	53	56	54	45	47	59	54	59	59

**Table 2:** Eruption status wise distribution of 1039 right and left sides mandibular third molar sites in 578 patients.

Clinical Finding	Group I		Group II		Group III		Total		*ℵ* ^2^-value	Grand Total *n* (%)
	Right *n* (%)	Left *n* (%)	Right *n* (%)	Left *n* (%)	Right *n* (%)	Left *n* (%)	Right *n* (%)	Left *n* (%)		
Partially Erupted	216 (20.78)	216 (20.78)	168 (16.16)	180 (17.32)	00 (0.00)	00 (0.00)	384 (30.95)	396 (38.11)	0.66 NS *P* > .05	780 (75.07)

Not Erupted/ Missing/Under developed	37 (3.56)	51 (4.90)	43 (41.38)	40 (3.84)	42 (4.04)	46 (4.42)	122 (11.74)	137 (13.18)	0.43 NS *P* > .05	259 (24.92)

Total *n* (%)	253 (24.35)	267 (25.69)	211 (20.30)	220 (21.17)	42 (4.04)	46 (4.42)	506 (48.70)	533 (51.29)		1039 (100)

*ℵ* ^2^-value							146.2 *P* < .0001 S *P* < .05	145.6 *P* < .0001 S *P* < .05

**Table 3:** Radiographic angulation wise distribution of total right and left sides impacted mandibular third molars.

Angulation	Group I		Group II		Group III		Total		*ℵ* ^2^-value	Grand Total *n* (%)
	Right *n* (%)	Left *n* (%)	Right *n* (%)	Left *n* (%)	Right *n* (%)	Left *n* (%)	Right *n* (%)	Left *n* (%)		
Mesioangular	87 (8.80)	84 (8.50)	73 (7.38)	56 (5.66)	27 (2.73)	28 (2.83)	187 (18.92)	168 (17.00)	0.52 NS *P* > .05	355 (35.93)

Distoangular	41 (4.14)	36 (3.64)	32 (3.23)	38 (3.84)	1 (0.10)	3 (0.30)	74 (7.48)	77 (7.79)	0.41 NS *P* > .05	151 (15.28)

Horizontal	19 (1.92)	27 (2.73)	14 (1.41)	20 (2.02)	6 (0.60)	7 (0.70)	39 (3.94)	54 (5.46)	0.94 NS *P* > .05	93 (9.41)

Vertical	92 (9.31)	107 (10.82)	84 (8.50)	97 (9.81)	4 (0.40)	5 (0.50)	180 (18.21)	209 (21.15)	0.99 NS *P* > .05	389 (39.37)

Total *n* (%)	239 (24.19)	254 (25.70)	203 (20.54)	211 (21.35)	38 (3.84)	43 (4.35)	480 (48.58)	508 (51.41)		988 (100)

*ℵ* ^2^-value							26.81 0.0002 S *P* < .05	31.18 *P* < .0001 S *P* < .05

**Table 4:** Radiographic level of eruption-wise distribution of total right and left sides impacted mandibular third molars.

Levels	Group I		Group II		Group III		Total		*ℵ* ^2^-value	Grand Total *n* (%)
	Right *n* (%)	Left *n* (%)	Right *n* (%)	Left *n* (%)	Right *n* (%)	Left *n* (%)	Right *n* (%)	Left *n* (%)		
Level A	154 (15.58)	164 (16.59)	130 (13.15)	147 (14.87)	7 (0.70)	9 (0.91)	291 (29.45)	320 (32.38)	0.89 NS *P* > .05	611 (61.84)

Level B	81 (8.19)	79 (7.99)	60 (6.07)	57 (5.76)	22 (2.22)	22 (2.22)	163 (16.49)	158 (15.99)	0.98 NS *P* > .05	321 (32.48)

Level C	4 (0.40)	11 (1.11)	13 (1.31)	7 (0.70)	9 (0.91)	12 (1.21)	26 (2.63)	30 (3.03)	0.07 NS *P* > .05	56 (5.66)

Total *n* (%)	239 (24.19)	254 (25.70)	203 (20.54)	211 (21.35)	38 (3.84)	43 (4.35)	480 (48.58)	508 (51.41)		988 (100)

*ℵ* ^2^-value							49.65 *P* < .0001 S *P* < .05	59.07 *P* < .0001 S *P* < .05

**Table 5:** Radiographic third molar space-wise distribution of total right and left sides impacted mandibular third molars.

Third Molar Space	Group I		Group II		Group III		Total		*ℵ* ^2^-value	Grand Total *n* (%)
	Right *n* (%)	Left *n* (%)	Right *n* (%)	Left *n* (%)	Right *n* (%)	Left *n* (%)	Right *n* (%)	Left *n* (%)		
Class I	60 (6.07)	32 (3.23)	49 (4.95)	27 (2.73)	10 (1.01)	2 (0.20)	119 (12.04)	61 (6.17)	0.42 NS *P* > .05	180 (18.21)

Class II	179 (18.11)	220 (22.26)	152 (15.38)	182 (18.42)	21 (2.12)	33 (3.34)	352 (35.62)	435 (44.02)	0.66 NS *P* > .05	787 (79.65)

Class III	0 (0.00)	2 (0.20)	2 (0.20)	2 (0.20)	7 (0.70)	8 (0.80)	9 (0.91)	12 (1.21)	0.43 NS *P* > .05	21 (2.12)

Total *n* (%)	239 (24.19)	254 (25.70)	203 (20.54)	211 (21.35)	38 (3.84)	43 (4.35)	480 (48.58)	508 (51.41)		988 (100)

*ℵ* ^2^-value							62.74 *P* < .0001 S *P* < .05	55.03 *P* < .0001 S *P* < .05

**Table 6:** Radiographic inferior alveolar canal relation-wise distribution of total right and left sides impacted mandibular third molars (True Relation and False Relation).

Inferior Alveolar Canal Relation	Group I		Group II		Group III		Total		*ℵ* ^2^-value	Grand Total *n* (%)
	Right *n* (%)	Left *n* (%)	Right *n* (%)	Left *n* (%)	Right *n* (%)	Left *n* (%)	Right *n* (%)	Left *n* (%)		
True Relation										

Notching	26 (2.63)	29 (2.93)	35 (3.54)	22 (2.22)	6 (0.60)	6 (0.60)	67 (6.78)	57 (5.76)	0.31 NS *P* > .05	124 (12.55)

Grooving	15 (1.51)	20 (2.02)	7 (0.70)	14 (1.41)	5 (0.50)	6 (0.60)	27 (2.73)	40 (4.04)	0.72 NS *P* > .05	67 (6.78)

Perforation	3 (0.30)	3 (0.30)	2 (0.20)	5 (0.50)	3 (0.30)	4 (0.40)	8 (0.80)	12 (1.21)	0.72 NS *P* > .05	20 (2.02)

								Total true relations		211 (21.35)

False Relation										

Superimposed	97 (9.81)	105 (10.62)	81 (8.19)	99 (10.02)	16 (1.61)	15 (1.51)	194 (19.63)	219 (22.16)	0.72 NS *P* > .05	413 (41.80)

Adjacent	71 (7.18)	76 (7.69)	61 (6.17)	51 (5.16)	7 (0.70)	9 (0.91)	139 (14.06)	136 (13.76)	0.52 NS *P* > .05	275 (27.83)

None	27 (2.73)	21 (2.12)	17 (1.72)	20 (2.02)	1 (0.10)	3 (0.30)	45 (4.55)	44 (4.45)	0.37 NS *P* > .05	89 (9.00)

								Total false relations		777 (78.64)

Total *n* (%)	239 (24.19)	254 (25.70)	203 (20.54)	211 (21.35)	38 (3.84)	43 (4.35)	480 (48.58)	508 (51.41)		988 (100)

*ℵ* ^2^-value							12.49 *P* = .0019 S *P* < .05	6.99 *P* = .03 S *P* < .05		
